# Discovering missing reactions of metabolic networks by using gene co-expression data

**DOI:** 10.1038/srep41774

**Published:** 2017-02-02

**Authors:** Zhaleh Hosseini, Sayed-Amir Marashi

**Affiliations:** 1 Department of Biotechnology, College of science, University of Tehran, Tehran, Iran

## Abstract

Flux coupling analysis is a computational method which is able to explain co-expression of metabolic genes by analyzing the topological structure of a metabolic network. It has been suggested that if genes in two seemingly fully-coupled reactions are not highly co-expressed, then these two reactions are not fully coupled in reality, and hence, there is a gap or missing reaction in the network. Here, we present GAUGE as a novel approach for gap filling of metabolic networks, which is a two-step algorithm based on a mixed integer linear programming formulation. In GAUGE, the discrepancies between experimental co-expression data and predicted flux coupling relations is minimized by adding a minimum number of reactions to the network. We show that GAUGE is able to predict missing reactions of *E. coli* metabolism that are not detectable by other popular gap filling approaches. We propose that our algorithm may be used as a complementary strategy for the gap filling problem of metabolic networks. Since GAUGE relies only on gene expression data, it can be potentially useful for exploring missing reactions in the metabolism of non-model organisms, which are often poorly characterized, cannot grow in the laboratory, and lack genetic tools for generating knockouts.

## Genome-scale metabolic networks

Metabolic network models are among the best-studied biological networks^1^,^2^. The prevailing availability of high-throughput ‘omics’ datasets, such as genomics, transcriptomics and metabolomics data, has greatly facilitated (semi-) automated metabolic network reconstruction^3^,^4^,^5^,^6^. Furthermore, recent advances in the development of constraint-based methods have made these models suitable tools for computational analysis of biological systems^7^,^8^. However, completeness of metabolic network models has remained a major challenge^9^, as a model with missing information may not have sufficient accuracy to be used in biotechnological and biomedical studies^2^. Even the most comprehensive metabolic network models contain gaps, due to our imperfect knowledge about metabolic processes^10^,^11^.

## Gap analysis: gap finding and gap filling

So far, several gap analysis algorithms have been developed to be applied in metabolic network reconstruction. Table 1 shows a summary of these methods. Typically, gap analysis methods consist of two main steps. The first step is to find ‘imperfections’ of the network. A group of these methods, like GapFind/GapFill and FastGapFill, detect topological flaws of the network such as blocked reactions and dead-end metabolites. Other gap finding methods try to find the inconsistencies between model predictions and experimental data such as growth profiles on different media, fluxome data, or gene essentiality data. The second step is to add a set of reactions to the network to resolve the observed flaws and inconsistencies. The objective of most of the gap analysis methods is to minimize the number of reactions which should be added to the model so that model modifications be as minimal as possible. This set of reactions can be determined by optimization programming (Linear Programming, Mixed Integer Linear Programming or Quadratic Programming) or by heuristic approaches (see Table 1).

As it is shown in Table 1, different kinds of experimental data have been used for gap filling of metabolic networks so far. For instance, SMILEY uses the growth profile of an organism on different carbon or nitrogen sources in order to find inconsistencies between model predictions and experimental results^12^. While this data can be readily available for many bacteria (by means of Biolog™ microplates, for example) there is not sufficient data of growth profiles for eukaryotic organisms, which restricts the use of this method for gap filling. Another well-known method, GrowMatch, makes use of gene essentiality data for analysis of gaps^13^. Although correct predictions of essential genes is very important for a metabolic network model to be considered reliable, identifying essential genes experimentally, is a very hard and time-consuming task, and additionally, requires specific genetic tools for generating knockouts. Therefore, this type of data may not be available for many organisms. This is also true about ^13^C labeling data or metabolomics data which are required for OMNI^14^ and minimal extension^15^ methods, respectively. The four last methods in Table 1 usually take a draft metabolic network that cannot produce biomass, and try to add minimum number of reactions to the model, such that biomass producing reaction can carry flux^16^,^17^,^18^,^19^. In the present study we aim to use gene expression data for finding the gaps. Today, transcriptomes are relatively easy to obtain, which makes them attractive sources of information for being used in gap analysis of metabolic networks.

## Flux coupling and gene co-expression

Flux coupling analysis (FCA) is a computational method to determine, for each pair of reactions *i* and *j* in a metabolic network, how their fluxes (*v*_*i*_ and *v*_*j*_) depend on each other^20^. Two reactions *i* and *j* are “fully coupled” if they always have proportional flux values, *v*_*i*_ = *cv*_*j*_, where *c* is a constant indicating the ratio of *v*_*i*_ and *v*_*j*_. If zero flux through one reaction, *v*_*i*_ = 0, always implies zero flux through the other reaction, *v*_*j*_ = 0 (but not vice versa), *j* is said to be “directionally coupled” to *i*. If two reactions are not flux-coupled, they are defined to be “uncoupled”^20^. It has been previously shown that those genes that encode fully coupled reactions show higher levels of co-expression compared to other genes^21^. Later, it was suggested that flux coupling relations may change considerably when the network becomes more complete, i.e. reactions are added to the network^22^. More precisely, it was shown that with a more complete network, the coupling relations will become more consistent with the experimental data of gene co-expressions^22^.

## GAUGE: A novel Gap Analysis method by Using Gene Expression data

In this work, we present a novel gap analysis method, GAUGE, which uses FCA of metabolic networks together with the publicly available gene expression data, in order to propose a strategy for gap finding and gap filling. It has been previously suggested that existence of a pair of fully coupled reactions with (nearly) uncorrelated gene expression suggests a gap in the network^22^. In other words, we hypothesize that such reactions must be directionally coupled or uncoupled in the complete network.

As a test case, we try to analyze the gaps in the genome-scale metabolic model of *E. coli*. The goal of our method is to improve a metabolic network such that there is maximum consistency between experimental gene expression data and theoretical flux coupling relationships. For this purpose, we first find gaps by identifying pairs of fully coupled reactions with “low” gene co-expression. Then, in a two-step algorithm based on mixed integer linear programming (MILP) formulation, we try to minimize the inconsistencies by adding a minimum number of reactions from a dataset of all known reactions to the network.

## Methods

Suppose that we have an incomplete metabolic network model with known gene-protein-reaction relationships. The goal of GAUGE is to suggest extra reactions whose addition to the network makes gene co-expression relations become more consistent with flux coupling relations. Here we present a brief description of this method and the data we used.

### Metabolic network model

The metabolic network model of *E. coli, i*JR904, which was reconstructed in 2003^23^ was used as the model. This model includes 904 genes and 1075 reactions, and therefore, includes a considerable number of gaps compared to our current knowledge of *E. coli* metabolism.

### Gene co-expression dataset

Dataset of gene co-expressions of *E. coli* were obtained from a previous study^21^. This dataset is obtained by combining *E. coli* gene expression data which had been available from Stanford database^24^ and ASAP database^25^. These expression data are from *E. coli* K12 growing under different conditions, such as different nutrient sources, mutations or environmental perturbations. Then, for each pair of genes in the dataset the Pearson correlation coefficient of gene expressions was computed.

### Universal dataset of reactions

The universal dataset of reactions was obtained from KEGG^26^. The initial version of the dataset comprised of 10882 reactions. From this set, we excluded those reactions which have the same metabolite as both a substrate and a product. In addition, identical reactions which have the same metabolites with different names were also manually excluded from the dataset. The final version of this dataset of reactions, which will be referred to as the “universal” dataset, includes 9587 reactions. If addition of reactions from KEGG could not resolve some of the inconsistencies, we use another universal dataset which is the set of exchange reactions for all of the metabolites in the model.

### Calculating gene coupling relations

**Figure 1:**
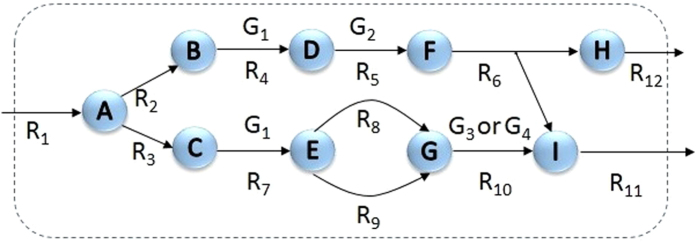
Difference between gene coupling and flux coupling. R_4_ and R_5_ are fully coupled reaction pairs. However, their associated genes, G_1_ and G_2_, are not fully coupled since G_1_ can also code for R_7_. As another example, R_7_ and R_10_ are fully coupled reactions. However, gene associated to R_7_ (G_1_) is not coupled to any of the genes associated to R_10_ (G_3_ and G_4_). This is because there is an “or” relationship between G_3_ and G_4_.

In the first step, gene pairs like (*g*_1_, *g*_2_) are found such that deletion of *g*_1_ inactivates all the reactions associated with *g*_2_, and vice versa. We require to find such gene pairs since our experimental data is for the co-expression of genes, not reactions. For this purpose, the following procedure is performed for every pair of metabolic genes in the model. First, *g*_1_ is removed from the model. Then, those reactions that cannot carry nonzero flux after the removal of this gene are identified. If all of the reactions that are associated with *g*_2_ are inactivated, then *g*_2_ is said to be *coupled* to *g*_1_. If *g*_1_ is coupled to *g*_2_ and vice versa, then we say that *g*_1_ and *g*_2_ are *fully coupled*. This procedure ensures that two genes entirely depend on the function of each other, and that they do not exhibit multiple functions which would justify independent gene expression. Figure 1 is a simple network which shows the difference between coupling of genes and reactions. As it is indicated, R_4_ and R_5_ are fully coupled reactions. However, according to the above procedure their corresponding genes, G_1_ and G_2_, are not fully coupled. This is because G_1_ is also associated to R_7_ and its function is independent of function of G_2_. Furthermore, R_7_ and R_10_ are also fully coupled reactions. However, since there is an “or” relationship between R_10_ genes, G_1_ in not dependent to any of them. We computed gene coupling relations for all of the gene pairs in the model.

### FCA for finding inconsistencies

As a preprocessing step, the biomass producing reaction is removed from the model in order to avoid a large set of fluxes to be detected as fully coupled^20^,^27^. Consequently, for each biomass component that could not be exported from the model, an export reaction was added. Therefore, all biomass components were allowed to be exported independently.

In the next step, flux coupling relations are calculated for every pair of reactions using F2C2^28^. Then, from the list of coupled gene pairs (previous section), those pairs that are linked to at least one pair of fully coupled reactions are selected. Now, for each of these gene pairs, if the gene expression values are uncorrelated based on the wet-lab experimental data, i.e., the Pearson correlation coefficient of the gene expression values are found to be below a certain threshold, the corresponding fully coupled reaction pairs are labeled as inconsistent. Such cases are considered as potential candidates for gap filling.

### MILP formulation

A two-step MILP formulation is used for resolving the discrepancies observed above. The inputs of the algorithm are: (i) the reaction pairs identified as discrepancy candidates; and (ii) the universal dataset of metabolic reactions. The goal is to add the smallest possible number of reactions from the universal dataset to the network, such that the highest possible number of inconsistencies are resolved.

We assume two consecutive steps for fixing model gaps. First, we assess whether addition of new reactions from the KEGG dataset or changing the reversibility type of irreversible reactions can change the coupling type of the candidate reaction pairs. If some of the discrepancies cannot be resolved by this way, we then check whether the addition of exchange reactions can fix the model gaps.

### MILP formulation of the first step

In the first step, the algorithm takes *S, U* and *U*_*e*_ matrices, and *L, H, R, D, D*_*e*_ and *IRR* sets as inputs which are defined as follows. *S, U* and *U*_*e*_ contain the stoichiometric coefficients for the reactions in the original model, the “universal” dataset of KEGG reactions and the universal dataset of exchange reactions, respectively. *L* is the set of fully coupled reaction pairs whose corresponding genes are uncorrelated, i.e., their Pearson correlation coefficient values are below a certain threshold. Similarly, *H*, is the set of fully coupled reaction pairs whose corresponding genes are highly correlated, i.e., their Pearson correlation coefficient values are above a certain threshold. In this study 0.2 and 0.8 are chosen as thresholds for reactions in *L* and *H*, respectively. *R, D* and *D*_*e*_ are set of the reactions of the original model, the universal dataset of KEGG reactions and the universal dataset of exchange reactions, respectively. Finally, *IRR* is the set of irreversible reactions in the original model.

By imposing the following constraints, the output of the algorithm would be the maximum number of the inconsistencies that can be resolved between gene co-expression and flux coupling relations. Note that first we use the *U* matrix to select reactions for addition to the model from KEGG dataset. If some of the inconsistencies could not be resolved this way, we run the MILP again with *U*_*e*_ and *D*_*e*_ as inputs, to select the candidate reactions from the dataset of exchange reactions.


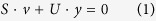


Constraint (1) imposes a stoichiometric mass balance on all of the metabolites, where *v* and *y* are vectors that contain the fluxes through reactions of the original model and the universal dataset, respectively.

To count the number of resolved inconsistencies, we need another constraint:


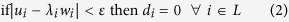


In constraint (2), every *i* represents a pair of fully coupled reactions with fluxes *u*_*i*_ and *w*_*i*_, whose corresponding genes are uncorrelated, and *λ*_*i*_ = *u*_*i*_/*w*_*i*_ is a constant. Vector *d* is a binary vector such that *d*_*i*_ = 1 if the two reactions of the reaction pair *i* are not fully coupled anymore after the network completion, and *d*_*i*_ = 0 otherwise. Here *ɛ* is used to avoid numerical errors of the MILP solver. In this work, we assume that *ɛ* *=* 10^–6^.

Note that Constraint (2) is not presented in the form of a linear constraint. However, it can be shown that application of the following four linear constraints is equivalent to Constraint (2):


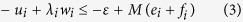



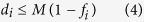



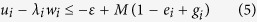



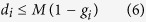


where *e, f* and *g* are binary vectors and *M* is a sufficiently large value.

It is also necessary to ensure that all of the reaction pairs in *H* remain fully coupled after addition of reactions from the dataset. Constraint (7) is used for this purpose:


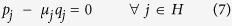


In Constraint (7), every *j* represents a fully coupled reaction pair with fluxes *p*_*j*_ and *q*_*j*_. Similar to Constraint (2), *μ*_*j*_ = *p*_*j*_/*q*_*j*_ is a constant.

Whenever the capacity constraints are known, it is important to include them in the MILP:


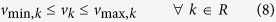



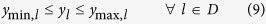


Equations (8) and (9) constrain the fluxes of the reactions in the original model and the dataset between the specified lower bounds and upper bounds. Since we are looking for the possibility of changing the reversibility of some reactions, all of the reactions in the original model are considered reversible and have negative lower bounds.

By applying the above mentioned constraints, it is possible to find solutions in which inconsistent reaction pairs of *L* are not fully coupled anymore. To obtain the best solution, the following objective function is used:


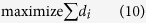


Using this objective function one can determine the maximum number of inconsistencies that are resolved using the MILP.

### MILP formulation of the second step

When the above-mentioned MILP is solved, the maximum number of inconsistencies which can be resolved, say Z^*^, will be determined. Now, in the second step of GAUGE, the goal is to minimize the number of reactions that should be added to the network or made reversible to resolve the inconsistencies. The inputs and constraints of the MILP of the second step is the same as the first step, with four additional constraints (11 to 14):


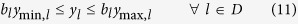


In Constraint (11) binary variables *b*_*l*_ are used for counting the number of added reactions from the universal dataset. Here, *b*_*l*_ = 1 if the corresponding reactions in the dataset carries nonzero flux. In other words, by imposing this constraint, *b*_*l*_ = 1 if *y*_*l*_ > 0.

We also consider the possibility of making some reactions reversible, for resolving model inconsistencies. To count the number of reactions which are made reversible, the following constraints are added:


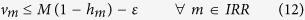



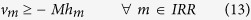


Constraints 12 and 13 ensure that if an originally irreversible reaction takes a negative flux after network completion, the value of binary variable, *h*_*m*_, is set to 1. Again M is a large value and *ɛ* = 10^−6^.

Now, to ensure that maximum possible number of inconsistencies are resolved in the second step, we apply the following constraint:


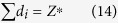


Constraint (14) fixes the sum of elements in *d* to its maximum value obtained by solving the first MILP.

Finally, to ensure that minimum number of reactions are added to the network or made reversible to resolve the inconsistencies, we use the following objective function for the second MILP:


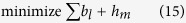


Altogether, by solving this MILP, we will find the minimum number of modifications which should be made to the network to maximally resolve the network inconsistencies.

### Alternative solutions

For calculating alternative solutions, additional constraints were iteratively added to the second MILP and the problem is solved again. This procedure is repeated until all of the optimal solutions are found. The additional constraints are as follows:


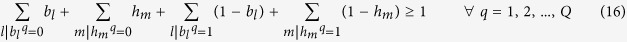


where *Q* is the number of solutions that are already identified. This constraint ensures that every solution vector differs with the previously found solutions by at least one element.

We should emphasize that if some of the network inconsistencies are not resolved by adding reactions from KEGG or making some reactions reversible, addition of exchange reactions are considered. For this purpose, the following changes are made to the above-mentioned MILPs: Instead of *U* and *D, U*_*e*_ and *D*_*e*_ are used as inputs; Constraints 12 and 13 are not considered anymore; and all of the irreversible reactions of the original model will take the lower bounds of zero.

### Code availability

The code is implemented for COBRA toolbox and is available here https://github.com/zhalehhosseini/GAUGE

### GapFind/Gapfill, Smiley and GrowMatch methods

GapFind/Gapfill^29^, Smiley^12^ and GrowMatch^13^ were used for gap filling and comparison with GAUGE. Single gene knockout data for GrowMatch and growth profiles for Smiley were obtained from EcoCyc database^30^. For GapFind, the COBRA Toolbox implementation was used^31^. For Smiley, GrowMatch and GapFill we used in-house implementations of these algorithms.

## Results and Discussion

### Application of GAUGE: *E. coli* as a case study

Here, we describe the use of GAUGE to resolve the inconsistencies between experimental gene co-expression data and *in silico* flux coupling relationships of the *i*JR904 metabolic network of *E. coli*^23^. Characterizing a pair of metabolic genes as “correlated” or “uncorrelated” pair requires a cutoff for computed values of co-expressions. In this study we define *L* as the set of reaction pairs with absolute correlation coefficients of less than 0.2 and *H* as a set of reaction pairs with absolute correlation coefficients of greater than 0.8. The goal of GAUGE is to change the coupling type of the reaction pairs in *L* while keeping the coupling type of the reaction pairs in *H* unchanged. For the *i*JR904 metabolic model, *L* and *H* include 134 and 41 reaction pairs, respectively. The existence of each inconsistent reaction pair in *L* implies that there are some missing reaction(s) in the network. Addition of such reactions to the model will change the coupling type of the reaction pair in *L* from full coupling to other types of flux (un) coupling relations. We used GAUGE to resolve the inconsistencies by adding reactions from KEGG and changing the reversibility type of reactions or by adding exchange reactions to the model.

### Computing globally optimal solutions for resolving the inconsistencies

**Figure 2:**
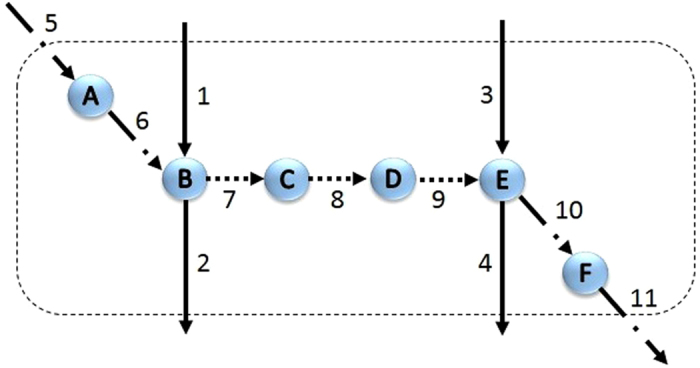
Difference between resolving inconsistent cases one by one or all at once. If inconsistent fully coupled reactions (1 and 2) and (3 and 4) are resolved once at a time, reactions 5, 6, 10 and 11 are chosen for addition to the model. However, if they needed to be resolved together, reactions 7, 8 and 9 are the minimal set of reactions for addition to the network.

Resolving the inconsistencies can be done by two different approaches: all inconsistencies can be resolved at once or they can be resolved once at a time. Clearly, these two approaches may result in different set of predicted gap-filling reactions. Figure 2 is a simple example that shows this difference. In this figure, suppose that (1 and 2) and (3 and 4) are inconsistent reaction pairs. Trying to resolve these inconsistencies one by one, results in the addition of reactions 5 and 6 for pair (1 and 2) and reactions 10 and 11 for pair (3 and 4). However, if these cases are resolved together, reactions 7, 8 and 9 are the minimal set of reactions that are needed for addition to the network. In order to have a globally minimal solution, we input the inconsistent reaction pairs all at once to the first step of the algorithm to calculate the maximum number of these cases that could be resolved. GAUGE identified consistency-returning suggestions for 132/134 pairs of *L*. Out of the 132 inconsistency cases, 54 cases were resolved by adding reactions from KEGG, 2 cases were resolved by forcing irreversible reactions to have flux in the backward direction, and the others by allowing the exchange of metabolites between extracellular space and cytoplasm. At minimum, addition of 31 KEGG reactions and 18 exchange reactions and changing the reversibility type of 1 reaction are needed to resolve the inconsistencies of these 132 cases. The detailed information about these results and the procedure of computing alternative solutions are described in the Supplementary file.

Here we discuss a few examples of resolved inconsistencies by GAUGE predictions that evidence from databases or literature exist for their presence in *E. coli*.

### Methylglyoxal metabolism

**Figure 3:**
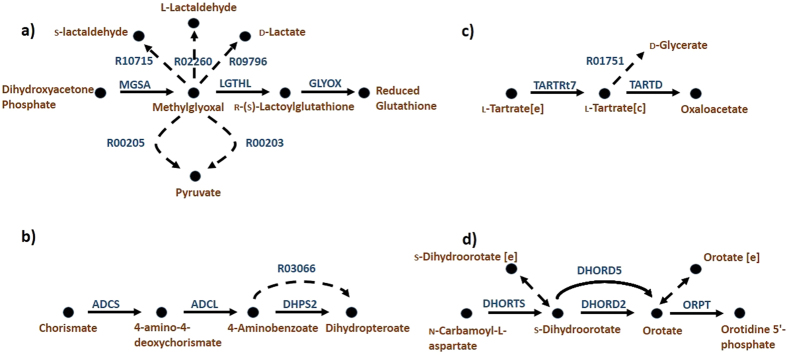
Four examples of correct predictions of GAUGE. (**a**) MGSA and GLYOX are inconsistent reaction pairs in *i*JR904. Reactions shown in dashed-lines, (R09796, R02260, R10715, R00205 and R00203) are GAUGE predictions. R09796, R02260 and R00203 are positively validated. (**b**) ADCS and DHPS2 are inconsistent reaction pairs. R03066 is GAUGE prediction which its catalyzing enzyme is encoded in *E. coli* genome. (**c**) TARTRt7 and TARTD are inconsistent reaction pairs. GAUGE predicts addition of R01751 which its corresponding enzyme is encoded in *E. coli* genome. [e] and [c] are used for extracellular and cytosolic metabolites, respectively. (**d**) DHORTS and ORPT are inconsistent reaction pairs. GAUGE predicts the addition of exchange reactions of Orotate or s-Dihydroorotate. The exchange reaction for Orotate is also proved to exist in *E. coli*.

Figure 3a shows a part of Methylglyoxal metabolism. Reactions GLYOX and MGSA are two reactions in this pathway which are fully coupled in *i*JR904 with Pearson correlation coefficients of less than 0.2. GAUGE predicts 5 separate reactions to resolve the inconsistency in this case. Interestingly, for two of these reactions (R02260 and R09796) evidence can be found for their presence in *E. coli*^32^,^33^,^34^. In addition, one other reaction, R00203, is catalyzed by an enzyme which is known to be encoded in the *E. coli* K12 genome. More precisely, R00203 is catalyzed by lactaldehyde dehydrogenase (E.C. number 1.2.1.22). This enzyme is encoded in *E. coli* genome and catalyzes the conversion of l-lactaldehyde to l-lactate. It is also shown that this enzyme catalyzes the conversion of methylglyoxal to pyruvate (reaction R00203) in *E. coli*^35^. However, the K_m_ for this conversion is higher compared to the conversion of l-lactaldehyde to l-lactate. It should be noted that R00203 and R00205 in the Figure, differ in the cofactor used by their catalyzing enzymes.

### Folate metabolism

Figure 3b shows part of the Folate metabolism pathway. In this Figure, ADCS and DHPS2 are inconsistent reaction pairs. GAUGE predicts R03066 to be added to the model. This reaction is catalyzed by dihydropteroate synthase (E.C. number 2.5.1.15) which is encoded by a gene present in *E. coli* genome (b3177). Additionally, based on the KEGG database this reaction is present in folate metabolism pathway of *E. coli* K12.

### Tartrate metabolism

TARTD and TARTRt7, as another inconsistent pair found by GAUGE, are shown in Fig. 3c. GAUGE predicts the addition of R01751, which is catalyzed by tartrate decarboxylase (E.C. number 4.1.1.73). d-malate oxidase is an enzyme with the same E.C. number which is encoded by b1800 gene in *E. coli* model, and interestingly, this enzyme is also annotated as “putative tartrate dehydrogenase” in *E. coli*.

### Purine and pyrimidine biosynthesis

DHORTS and ORPT are inconsistent reaction pairs in Fig. 3d. GAUGE could not identify any reactions in KEGG to resolve the inconsistency of this case. In addition, no change in reversibility types can resolve it. However, GAUGE predicts the addition of exchange reactions for orotate or s-dihydroorotate. The gene for transporting orotate to the cell is also known to be present in *E. coli*^36^.

### Comparison of GAUGE results with other gap filling methods

We have run GapFind/GapFill^29^, Smiley^12^ and GrowMatch^13^ algorithms on the same metabolic network, to compare their results with GAUGE. We should emphasize here that there are no standard benchmark for comparing gap analysis methods. Each method uses different kind of inputs and searches for different types of gaps. In addition, false negativity, true negativity, or even false positivity cannot be defined for the results of gap analysis methods, since a comprehensive and perfect knowledge about the metabolism of organisms does not exist. Therefore, we can only try to compare the results of different gap analysis methods by searching for evidence for the reactions predicted by each method and calculating the frequency of supported predictions for each method.

GrowMatch solves two MILPs to add and remove reactions for resolving the NGG (*in silico* no growth vs. *in vivo* growth) and GNG (*in silico* growth vs. *in vivo* no growth) cases respectively. Since GAUGE only predicts reactions for being added to the model, only the MILP for resolving NGG cases was run to obtain comparable results. Altogether, 37 NGG cases were identified. Every NGG case was used separately and all of the alternative optimal solutions were calculated for each case. From these cases, 18 cases could be resolved using one of the three possible strategies, namely, addition of reactions from KEGG, changing irreversible reactions to reversible ones, and addition of exchange reactions. The total of 69 reactions were predicted for being added to the model or changing their reversibility type.

Smiley is a method that resolves the inconsistency between observed *in vivo* growth phenotypes and predicted *in silico* growth patterns. This algorithm uses information of growth profiles on different carbon and nitrogen sources as inputs and solves an MILP formulation to add minimum number of reactions to the model to resolve false negative model predictions. Reactions were selected from KEGG dataset or dataset of exchange reactions. Using Smiley, 34 false negatives were identified and 17 out of these 34 cases could be resolved. By calculating all alternative solutions, the algorithm predicted a total number of 55 reactions for gap filling.

GapFind/GapFill finds no-production metabolites in the model and add minimal set of reactions to restore the connectivity of these metabolites to the rest of the network. Using GapFind, 64 inconsistent metabolites were found in *i*JR904. From these cases, 63 cases could be resolved using one of the three possible strategies, namely, addition of reactions from KEGG, changing irreversible reactions to reversible ones, and addition of exchange reactions. This method predicts 84 reactions for addition to the model or changing the reversibility type.

Since all these algorithms resolve the inconsistency cases one by one, to obtain comparable results, we input each inconsistent reaction pair separately to GAUGE and identified all of the possible alternative optimal solutions for each case. GAUGE predicts 89 reactions as the candidates for being added to the model or being made reversible.

In the next step, the correctness of the predictions of each algorithm was validated by:

1. looking for the presence of a link between these reactions and a gene in *E. coli* genome annotations in KEGG database. In other words, if, according to the KEGG database, a gene from *E. coli* genome can code for the catalyzing enzyme of the predicted reaction, we suppose that this reaction can occur in this organism.
2. performing BLASTP against the *E. coli* K12 genome. More precisely, the best hits in the *E. coli* genome which have the BLASTP E value of less than 10^−20^ are considered as potential coding genes for the predicted enzyme activities in *E. coli*.

We also searched the literature to find evidence regarding the presence of enzyme activities in *E. coli* which are predicted by GAUGE. The detailed information about the predicted reactions by each method is presented in the Supplementary Tables S2, S3, S4 and S5.

**Figure 4:**
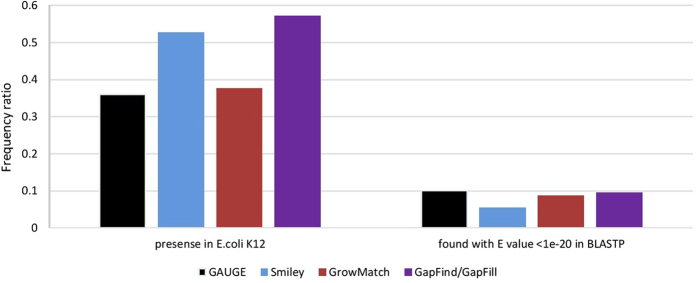
Comparison of the results predicted by GAUGE, Smiley, GrowMatch and GapFind/GapFill. The first category from the left is for reactions which are present in *E. coli* k12. The second category represents the reactions which evidence are found for them from BLASTP.

Figure 4 shows the percentage of correct predictions of each algorithm. As shown, GapFind/GapFill and Smiley have the most successful predictions. This observation is presumably due to the logic behind these algorithms. Smiley tries to correct the false negative predictions of the model grown on different media. When the cell can grow *in vivo* on a media, it must have the capability to convert the available nutrients to biomass precursors. Therefore, the failure of *in silico* prediction definitely implies missing reactions which leads to the precise predictions by Smiley. The same is true for GapFind/GapFill. It is not reasonable to have a metabolite in the cell with no production route and GapFind searches for these metabolites. Therefore, there is a high probability that its predictions are correct. The reason for less correctness of GrowMatch predictions may be that the presence of NGG cases are not necessarily because of missing reactions. For example, the reason may be that another isozyme which is missing from the model catalyzes the same reaction. In case of GAUGE, the algorithm was tested based on gene co-expression data obtained by Pearson correlation. Using more accurate and complete gene expression datasets, choosing different thresholds for the co-expression of genes, and the application of better correlation measures^37^ can potentially improve the predictions of GAUGE.

### “Orthogonality” of gap analysis methods

**Figure 5:**
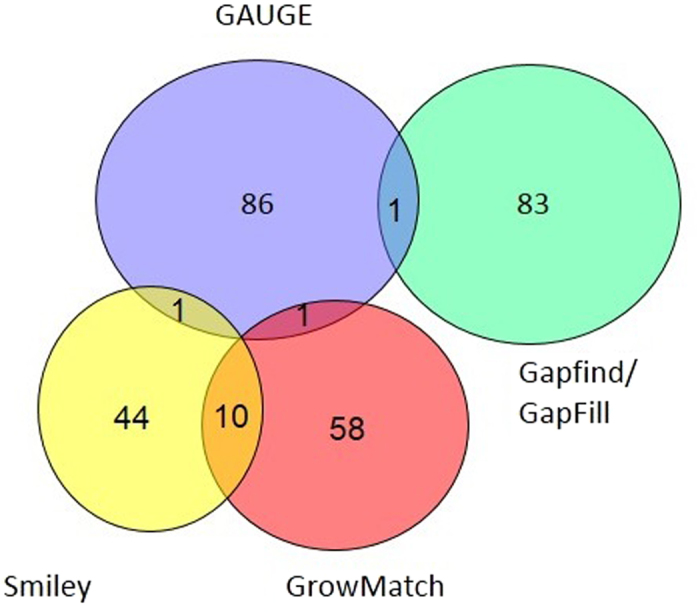
Venn diagram of reactions predicted by each method.

Figure 5 shows the Venn diagram of the number of gap-filling reactions predicted by each method. As shown, among the reactions predicted by GAUGE only three reactions are in common with the results of Smiley, GrowMatch or GapFind/GapFill. If only positively validated reactions are considered, there will be no common reactions between predictions of GAUGE with other methods (See Supplementary Figure S2). These results show that GAUGE can predict different sets of reactions for being added to the model during gap filling. This finding was expected, as the logic behind our method is principally different from other gap filling approaches. In other words, GAUGE can be used as a complementary strategy to the existing strategies for filling the gaps of metabolic networks.

We also investigated in which biological pathways, the predicted gap-filling reactions are involved. Only the reactions which were positively validated, are considered. The pathways for each method are shown in Supplementary Figure S3. As it is shown, there are some biological pathways that are only captured by one single method. For example, lipopolysaccharide metabolism, riboflavin metabolism, nitrogen metabolism, valine, luecine and isoleucine degradation, lysine biosynthesis, d-alanine metabolism, and synthesis and degradation of ketone bodies are pathways that are only identified by GAUGE. In other words, Smiley, GrowMatch and GapFind/GapFill are found to be unable to explore the missing reactions of these pathways. Identification of biological pathways that are unique to only one method shows that each gap analysis method examines specific parts of the metabolism that are not considered by other methods. This may be the result of the fact that each method looks for model errors from a particular point of view, and application of popular methods like Smiley, GrowMatch or GapFind/GapFill does not eliminate the necessity of application of GAUGE.

As a final note, it should be mentioned that although GAUGE is based on MILPs, in practice it works acceptably fast. For example, for *i*JR904 network, when inconsistencies are resolved one by one, the mean computation time of GAUGE is ~13 seconds on a PC. The computation time of resolving inconsistencies all at once is ~30 minutes.

### Robustness analysis of GAUGE

**Figure 6:**
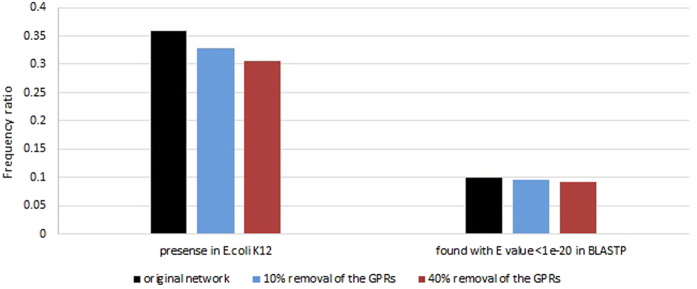
Comparison of the results predicted by GAUGE when 0, 10 and 40 percent of the reactions are removed from the network. The first category from the left is for reactions which are present in *E. coli* k12. The second category represents the reactions which evidence are found for them from BLASTP.

In order to investigate how sensitive GAUGE is to the lack of GPRs, we randomly removed some of the GPRs from the model and performed GAUGE on them. Two groups of 100 random networks were generated in which 10 and 40 percent of the reaction GPRs were removed respectively. Then for each network, gene coupling relations were calculated and inconsistent reaction pairs were identified. All of the alternative solutions were computed for resolving each inconsistency. The results are shown in Fig. 6. When we remove 40 percent of the reaction GPRs from the *E. coli* network, the accuracy of predictions decreases from 36 percent to about 30 percent. Therefore, GAUGE predictions is not significantly affected by the varying degrees of coverage of the GPRs. This 6 percent reduction in accuracy is probably due to the fact that by deletion of some GPRs some of the genes will become fully coupled to each other. GAUGE will mistakenly predict some reactions to be added to the model for resolving inconsistency of these cases.

Another method for robustness analysis of GAUGE, is to randomly remove reactions from the model and analyze what percentage of them could be returned back using GAUGE. In the Supplementary file, we explain that this analysis is not suitable for evaluating GAUGE, since there is not a high probability that removed reactions are associated to a fully coupled gene with low co-expression.

## Conclusion

In the present work we have developed a gap analysis method, GAUGE, to resolve the cases where *in silico* flux coupling relationships is not in agreement with experimental gene co-expression patterns. GAUGE resolves the inconsistencies by adding reactions from KEGG database, changing the reversibility type of reactions or allowing exchange of metabolites between cytoplasm and extracellular space. We tested GAUGE on *i*JR904 metabolic network model of *E. coli* as a model that we know contains a large number of gaps. We were able to find out missing reactions that may not be recognizable by other gap filling methods. Therefore, GAUGE can be used as an alternative and complementary strategy for gap filling of metabolic networks. Usually, those methods that use topological flaws of the network such as dead-end metabolites are preferred for gap filling, since these methods can be applied without the need for an experimental dataset. For instance, obtaining gene essentiality data for every gene in an organism is not a simple task and such data is not available for many organisms. A benefit of GAUGE is that it uses a type of experimental data which is readily available for many organisms. Another beneficial feature of GAUGE is that there is the possibility to find globally optimal solutions, instead of finding solutions to solve the inconsistencies case by case. This is the approach that is also considered in very recent study of Hartleb *et al*.^38^. In this study, the authors present GlobalFit, a bi-leveloptimization method, which identifies minimal set of model changes to achieve a model that can correctly predict all of the experimental data of growth and non-growth cases.

Here, we have validated our results by searching in the literature and databases and also by performing BLASTP to find genomic evidence of genes. It should be noted that there is a new version of *E. coli* metabolic network model, *i*JO1366, which is reconstructed in 2011^39^. We have also looked for the predicted reactions in this version of the model. Interestingly, some of these reactions are included in *i*JO1366. These reactions are presented in Supplementary Tables S2, S3, S4 and S5. We should note that in the universal dataset of reactions used in this study, all of the reactions are included without considering their directionalities. It is definitely a valuable analysis to compute the Gibbs free energy change for each reaction and see in which direction it will carry flux. However, this is not a necessary step in validation of our gap filling results, since addition of reactions in each of the two directions will resolve the identified gaps. More clearly, as it is shown in Fig. 2, addition of reactions 5 to 11 (in forward or reverse directions) will change the coupling type of reactions 1 and 2 and reactions 3 and 4 from fully coupled to directionally coupled. If one needs to add the predicted reactions of GAUGE to a metabolic network, the Gibbs free energy changes should necessarily be computed to know in which direction the reactions should be added.

One should note that there are not a large number of inconsistent reaction pairs in the model. As shown in Supplementary Figure S3, the majority of genes are involved in a low number of full coupling relations, while a large number of genes are not fully coupled to any other genes (not shown in the graph). In addition, Supplementary Figure S4 shows that those genes which are associated with larger number of reactions are generally involved in lower number of full coupling relations. Therefore, fully coupled gene pairs which are associated to fully coupled reaction pairs are not frequent in metabolic networks. However, the results presented here show that even in these situations, GAUGE can successfully predict the novel reactions for being added to the model. Another point is that, other inconsistencies may exist between experimental gene co-expressions and theoretical flux coupling relations. One such inconsistency is when a highly co-expressed gene pair is not associated to fully coupled reactions. However, in this case one cannot draw any conclusion about the incorrectness of the model. The high co-expression may exist, for example, for functionally related genes, while these genes should not necessarily be fully coupled. Another point is that if some specific biochemical pathways are activated in the cell, some genes may not be highly co-expressed anymore. The environmental conditions which activate these pathways may not be captured in the experimental gene expression data. Therefore, having highly co-expressed gene pairs with no fully coupled reactions do not mean that the model should be modified, e.g., reactions should be deleted from the model. Using more comprehensive gene expression data may decrease the number of such inconsistencies. Furthermore, as our results suggest, only certain gaps are found, and can be filled, based on gene expression data. Moreover, regulation of protein expression may occur at the post-transcriptional level, which again means that gene expression data might not be sufficient for a comprehensive gap finding. Despite these shortcomings, we show that GAUGE can be used in practice to find and fill the metabolic gaps, and its performance is comparable to the other well-known widely used gap filling tools. Therefore, it is relevant to use transcriptional level gene expression data for gap filling.

GAUGE is presented here as a potential strategy for gap analysis of metabolic networks that predict different sets of reactions for addition to the model. Several parameters can be adjusted for improving the predictive power of GAUGE. Setting different thresholds for low and high correlation coefficients are one such parameter. Additionally, instead of computing Pearson correlation coefficients of gene expressions, the Boolean version of expression values may be considered, i.e., expression values higher and lower than a certain cut-off are considered as expressed or not expressed, respectively. Then, the genes which are always expressed together can be identified and labeled as fully coupled gene pairs. Another possibility is to use other measures of correlation like mutual information, instead of Pearson correlation coefficients. The gene expression data can also have an important effect on the results predicted by GAUGE. As mentioned above, completeness of dataset can affect the correlation of gene expressions, which in turn may affect the inconsistencies found between experimental observations and model predictions. For obtaining an optimized version of GAUGE with more reliable results all of the above mentioned points should be taken into account.

One may also think of using protein abundance data, e.g., from proteomic databases, instead of gene co-expression data. However, we should note that protein abundance data may contain more noise, including more false negatives, compared to gene expression data, which in turn may result in more unreliable predictions. On the other hand, in the study of Notebaart *et al*.^21^ it is shown that there is a good correlation between gene co-expressions and flux coupling relations.

We should also note that, one way to improve the GAUGE predictions is to use BLAST-weighted dataset of reactions like strategies used recently^18^,^19^. This way, the presence of unrelated or orphan reactions may be reduced in possible solutions of GAUGE.

## Additional Information

**How to cite this article**: Hosseini, Z. and Marashi, S.-A. Discovering missing reactions of metabolic networks by using gene co-expression data. *Sci. Rep.* **7**, 41774; doi: 10.1038/srep41774 (2017).

**Publisher's note:** Springer Nature remains neutral with regard to jurisdictional claims in published maps and institutional affiliations.

## Supplementary Material

Supplementary File

## Figures and Tables

**Table 1:** Characteristics of gap analysis methods.

Method name	Type of data used for checking model inconsistency	Optimization Algorithm	Strategy
GapFind/GapFill^29^	dead-end metabolites	MILP	Minimizing added reactions
FastGapFill^40^	blocked reactions	LP/MILP	Minimizing added reactions
SMILEY^12^	growth phenotype data	MILP	Minimizing added reactions
OMNI^14^	fluxome data	MILP	Minimizing difference between measured and predicted fluxes + maximizing biomass production flux
GrowMatch^13^	gene essentiality data	MILP	Minimizing added reactions
minimalExtension^15^	ability of converting nutrients to target metabolites	Greedy	Minimizing added reactions
FBA-Gap^16^	growth capability	MILP	Minimizing added reactions
FastGapFilling^17^	growth capability	Heuristic approach using LP	Maximizing biomass flux + Minimizing added reactions
Likelihood-based gap filling^18^	growth capability	MILP	Weighted minimization of added reactions
BLAST-weighted gap filling^19^	growth capability	LP/QP	Weighted minimization of sum of the fluxes of reactions

Two first methods use topological flaws for checking model inconsistencies. The other methods look for inconsistencies between experimental results and model predictions.
