# Supplementary material for: Discovering missing reactions of metabolic networks by using gene co-expression data
Source: Sci Rep. 2017 Feb 2;7:41774. doi: 10.1038/srep41774 (PMC5288723; doi:10.1038/srep41774)
Supplement: Supplementary File [file srep41774-s1.pdf]

Discovering missing reactions of metabolic networks by using gene co-expression data (Supplementary information)

Zhaleh Hosseini [1] and Sayed-Amir Marashi [1,*]

[1]Department of Biotechnology, College of science, University of Tehran, Tehran, Iran.

*Corresponding Author: marashi@ut.ac.ir

1- Global Optimal Solutions of GAUGE for *i*JR904

In order to have a globally minimal solution, we input the inconsistent reaction pairs all at once to the first step of the algorithm described in the manuscript, to calculate the maximum number of these cases that could be resolved. GAUGE identified consistency-returning suggestions for 132/134 pairs of *L*. Changing the reversibility type of one reaction (row 50), addition of 31 reactions from KEGG (rows 1-31) and addition of 18 exchange reactions (row 32-49) are needed at minimum to resolve the inconsistencies of these 132 cases. As computing all of alternative solutions is a very time-consuming task, we pursued the following procedure to compute a subset of all of the possible alternative solutions. First, we used inconsistent reaction pairs once at a time and compute all of the optimal solutions for each case (all of the solutions with minimum number of added reactions). Then, we used the union of these predicted reactions as universal dataset. Finally, we used all the inconsistency cases at once as well as this new version of universal dataset as inputs to GAUGE and computed all of the optimal alternative solutions.
The total of 414720 alternative solutions were calculated using the second step of GAUGE. We tried to verify the predicted reactions by three strategies:
1) We looked for the presence of a link between these reactions and a gene in *E. coli* genome in KEGG database. If, according to the KEGG database, a gene from *E. coli* genome can code for the catalyzing enzyme of the predicted reaction we suppose that this reaction can occur in this organism.
2) We performed BLASTP against the *E. coli* K12. The best hits in the *E. coli* genome which have the BLASTP E value of less than 10^{-20} are considered as potential coding genes for the predicted enzyme activities in *E. coli*.
3) We also searched the literature and the Ecocyc database for possible evidence regarding the presence of predicted enzyme activities in *E. coli* strains.
Based on our validation results, we chose the best possible solutions, i.e., those with the most number of supported reactions. The summary of this result is presented in Table S1.
In the third column of the table, the three above-mentioned validations for each reaction are shown. For reactions with evidence number 1, the gene ID(s) in KEGG are also presented. For reactions with evidence number 2, the E value and gene ID of the best hit in BLASTP are shown. Finally, for other reactions which found in the literature, the corresponding references are presented.

Table S1. The best global optimal solution of GAUGE. These reactions are the most supported set of alternative solutions.

	**reaction**	**evidence**	
1	R01365	KEGG database	b2221/b2222
2	R00414*<br>R02707*	KEGG database<br>KEGG database	b3786<br>b3786
3	R05552	KEGG database	b1812/b3360
4	R03066	KEGG database	b3177
5	R04209	KEGG database	b0522
6	R05554	KEGG database	b0515
7	R02423	KEGG database	b0516
8	R00776	KEGG database	b0505
9	R09376	BLASTP	3×10^{-20} (b0414)
10	R09377	BLASTP	10^{-25} (b0058)
11	R00484		
12	R01395*<br>R07316*	KEGG database<br>KEGG database	b0032/b0033/b0323/b0521/ b2874<br>b1011
13	R03546	KEGG database	b0340
14	R07613	BLASTP	2×10^{-89} (b2379)
15	R00160		
16	R00550*<br>R00548*	Literature<br>KEGG database	[1]<br>b0980/b4055
17	R01098		
18	R02252	BLASTP	6×10^{-82} (b3081)
19	R01573	Literature	[2]
20	R01576	Literature	[3]
21	R03161		
22	R01623	KEGG database	[4,5]
23	R01580	Literature	[6,7]
24	R10715*	KEGG database	b3945

	R09796*<br>R00203*<br>R02260*	KEGG database<br>KEGG database<br>Literature	b1967<br>b1415<br>[8]
25	R01309	KEGG database	b0494/b3825
26	R02054*<br>R02053*	KEGG database<br>KEGG database	b3821<br>b3821
27	R03417*<br>R03416*	KEGG database<br>KEGG database	b0494/b3825<br>b0494/b3825
28	R07306		
29	R09374		
30	R03191	KEGG database	b3972
31	R01751	KEGG database	b1800
32	Lipa_ex	Ecocyc database	
33	Adphep_LD_ex		
34	LipidA_ex	Literature	[9,10]
35	LipidAds_ex		
36	U3hga_ex		
37	Db4p_ex		
38	Dhor_S_ex*<br>orot_ex*	-<br>Ecocyc database	
39	Cechddd_ex		
40	3dhq_ex		
41	Gmhep17bp_ex		
42	Gmhep1p_ex		
43	U3aga_ex		
44	Kdo2lipid4_ex*<br>Kdo2lipid4L_ex*	Ecocyc database<br>-	
45	Fcl_L_ex	Literature	[11]
46	Orot5p_ex		
47	Dmlz_ex		
48	Sl2a6o_ex*,<br>sl26da_ex*		
49	Uaccg_ex*,<br>uamr_ex*		
50	LPLIPA4*<br>LPLIPA5*<br>LPLIPA6*		

* reactions with a star mark in each cell can be used interchangeably

2- All of the reactions predicted by GAUGE, Smiley, Gapfind/Gapfill and GrowMatch

The following four tables show list of the reactions predicted by each method and the available evidence for them. In tables S2, S4 and S5 reactions in rows 87-89, 63-69 and 51-84 are irreversible reactions in *i*JR904 which are predicted to be reversible by GAUGE, GrowMatch and GapFind/GapFill, respectively. Column "Presence in *i*JO1366" indicates reactions which are included in *i*JO1366 [12], the newer version of the E. coli model. Column "*E. coli* genes in KEGG" shows genes from KEGG database which are linked to the predicted reactions. In case of exchange reactions or reactions which are predicted to be reversible, this column shows available evidence from Ecocyc database. Column "BLASTP E value and gene ID of best hit" shows E value of BLASTP together with the gene corresponding to the best hit in BLASTP against *E. coli* K12. Column "orphan reactions" shows predicted reactions which are orphan (with no known coding genes). For table S2, articles which have evidence about the occurrence of predicted reactions in *E. coli* are also presented. Finally, the last column in each table shows the KEGG pathways in which the reactions are involved.

Table S2. Predictions of GAUGE when inconsistencies are resolved one by one.

	rxn ID	Presence in	*E. coli* genes in	BLASTP E value	orphan	KEGG pathways

		*i*JO1366	KEGG or Ecocyc	and gene ID of best hit	reactions	
1	R01357			3e-53 (b4069)		Valine, leucine and isoleucine degradation<br>Butanoate metabolism
2	R00414 [13,14]		b3786			Amino sugar and nucleotide sugar metabolism
3	R05552 [15,16]		b1812/b3360			Folate biosynthesis
4	R03066 [17]		b3177			Folate biosynthesis
5	R04209 [18,19]		b0522			Purine metabolism
6	R02423 [20]		b0516			Purine metabolism
7	R05554 [21]		b0515			Purine metabolism
8	R00776 [22,23]		b0505			Purine metabolism
9	R09375					Riboflavin metabolism
10	R09377			1e-25 (b0058)		Riboflavin metabolism
11	R00484					Alanine, aspartate and glutamate metabolism
12	R07613			2e-89 (b2379)		Lysine biosynthesis
13	R00160					Riboflavin metabolism
14	R08574					Riboflavin metabolism
15	R10616 [24]			2e-26 (b0268)		Galactose metabolism
16	R06780					Phenylalanine metabolism
17	R01573 [2]				✓	
18	R01576 [3]				✓	
19	R03161					Fructose and mannose metabolism<br>Amino sugar and nucleotide sugar metabolism
20	R01098					Galactose metabolism
21	R01148			2e-25 (b3770)		D-Alanine metabolism
22	R10715 [25,26]		b3945			Propanoate metabolism
23	R01309		b0494/b3825			Glycerophospholipid metabolism
24	R02054		b3821			Glycerophospholipid metabolism
25	R03417 [27,28]	✓	b0494/b3825			Glycerophospholipid metabolism
26	R07306					Riboflavin metabolism
27	R09374					Riboflavin metabolism
28	R03191 [29]		b3972			Amino sugar and nucleotide sugar metabolism
29	R03036					Pantothenate and CoA biosynthesis
30	R01751		b1800			
31	R00410			1e-59 (b2221)		Synthesis and degradation of ketone bodies<br>Valine, leucine and isoleucine degradation<br>Butanoate metabolism
32	R02707 [30]		b3786			Amino sugar and nucleotide sugar metabolism
33	R01176			3e-67 (b4069)		Butanoate metabolism
34	R00550 [1]				✓	Riboflavin metabolism
35	R02252			6e-82 (b3081)		Phenylalanine metabolism

36	R01580 [6,7]				✓	Vitamin B6 metabolism
37	R09796 [31,32]	✓	b1967			Pyruvate metabolism
38	R02053		b3821			Glycerophospholipid metabolism
39	R03416		b0494/b3825			Glycerophospholipid metabolism
40	R10747					Carbapenem biosynthesis
41	R01365		b2221/b2222			Lysine degradation
42	R02706					
43	R00548 [33]		b0980/b4055			Aminobenzoate degradation
44	R05839				✓	Vitamin B6 metabolism
45	R00205				✓	Pyruvate metabolism
46	R01623 [4,5]		b0404			Pantothenate and CoA biosynthesis
47	R01358				✓	Butanoate metabolism
48	R09376			3e-20 (b0414)		Riboflavin metabolism
49	R00203 [34]		b1415			Pyruvate metabolism
50	R01395		b0032/b0033/b0323/ b0521/b2874			Nitrogen metabolism
51	R03546 [35,36]		b0340			Nitrogen metabolism
52	R02260 [8]	✓				Pyruvate metabolism Propanoate metabolism
53	R00279				✓	D-Glutamine and D-glutamate metabolism
54	R07316 [37]		b1011			Nitrogen metabolism
55	lipa_exchange	✓	present in Ecocyc			Lipopolysaccharide biosynthesis
56	kdo2lipid4L_exchange					Lipopolysaccharide biosynthesis
57	adphep-LD_exchange					Lipopolysaccharide biosynthesis
58	u23ga_exchange					Lipopolysaccharide biosynthesis
59	db4p_exchange					Riboflavin metabolism
60	dhor-S_exchange					Pyrimidine metabolism
61	cechddd_exchange					Phenylalanine metabolism
62	3dhq_exchange					Phenylalanine, tyrosine and tryptophan biosynthesis
63	gmhep17bp_exchange					Lipopolysaccharide biosynthesis
64	adphep-DD_exchange					Lipopolysaccharide biosynthesis
65	kdo2lipid4_exchange	✓	present in Ecocyc			Lipopolysaccharide biosynthesis
66	lipidAds_exchange					Lipopolysaccharide biosynthesis
67	lipidA_exchange		present in Ecocyc			Lipopolysaccharide biosynthesis
68	kdolipid4_exchange		present in Ecocyc			Lipopolysaccharide biosynthesis
69	fcl-L_exchange		present in Ecocyc			Fructose and mannose metabolism
70	gmhep1p_exchange					Lipopolysaccharide biosynthesis

71	ugmda_exchange					Lysine biosynthesis
72	orot5p_exchange					Pyrimidine metabolism
73	dmlz_exchange					Riboflavin metabolism
74	sl2a6o_exchange					Lysine biosynthesis
75	u3hga_exchange					Lipopolysaccharide biosynthesis
76	uamag_exchange					D-Glutamine and D-glutamate metabolism
77	u3aga_exchange					Lipopolysaccharide biosynthesis
78	uaccg_exchange					Amino sugar and nucleotide sugar metabolism
79	ugmd_exchange					Lysine biosynthesis
80	orot_exchange	✓	present in Ecocyc			Pyrimidine metabolism
81	lipidX_exchange					Lipopolysaccharide biosynthesis
82	sl26da_exchange					Lysine biosynthesis
83	uamr_exchange					D-Glutamine and D-glutamate metabolism<br>Amino sugar and nucleotide sugar metabolism
84	ckdo_exchange					Lipopolysaccharide biosynthesis
85	gmhep7p_exchange		present in Ecocyc			Lipopolysaccharide biosynthesis
86	uama_exchange					D-Glutamine and D-glutamate metabolism
87	LPLIPA4					Glycerophospholipid metabolism
88	LPLIPA5					Glycerophospholipid metabolism
89	LPLIPA6					Glycerophospholipid metabolism

Table S3. Predictions of Smiley.

	rxn ID	Presence in *i*JO1366	*E. coli* genes in KEGG or Ecocyc	BLASTP E value and gene ID of best hit	Orphan reactions	KEGG pathways
1	R06613					Pyrimidine metabolism
2	R07676			4e-45 (b3012)		Pentose and glucuronate interconversions<br>Ascorbate and aldarate metabolism
3	R01000					Propanoate metabolism
4	R01094					Galactose metabolism
5	R03034					Galactose metabolism
6	R01097					Galactose metabolism
7	R01791			2e-178 (b4239)		Starch and sucrose metabolism
8	R02108		b1927/b3571			Starch and sucrose metabolism
9	R00028		b0403/b3878			Starch and sucrose

						metabolism
10	R01678		b0344/b3076 /b3077			Galactose metabolism
11	R00947		b1002			Glycolysis / Gluconeogenesis
12	R00878		b3565			Fructose and mannose metabolism
13	R09995		b3431			Starch and sucrose metabolism
14	R01797		b3918			Glycerophospholipid metabolism
15	R02030		b0789/b1249			Glycerophospholipid metabolism
16	R01799		b0175/b1409			Glycerophospholipid metabolism
17	R02027					Glycerophospholipid metabolism
18	R02057					Glycerophospholipid metabolism
19	R02051					Glycerophospholipid metabolism
20	R07390		b0789/b1249			Glycerophospholipid metabolism
21	R01800		b2585			Glycine, serine and threonine metabolism Glycerophospholipid metabolism
22	R01801		b1912			Glycerophospholipid metabolism
23	R04176	✓			✓	
24	R01951					Fructose and mannose metabolism Amino sugar and nucleotide sugar metabolism
25	R04270					
26	R02274					Lysine degradation
27	R07265			2e-129 (b2662)		
28	R02546					Glyoxylate and dicarboxylate metabolism
29	R07680					Ascorbate and aldarate metabolism
30	R10565					Pentose and glucuronate interconversions
31	R01906	✓	b3903			Pentose and glucuronate interconversions
32	R01901	✓	b3580			Pentose and glucuronate interconversions
33	R09100					
34	R03161					Fructose and mannose metabolism<br>Amino sugar and nucleotide sugar metabolism
35	R00215		b1800			Butanoate metabolism
36	Alpha-Ketobutyric Acid_exchange		present in ecocyc			Glycine, serine and threonine metabolism<br>Cysteine and methionine metabolism<br>Valine, leucine and isoleucine biosynthesis<br>Propanoate metabolism<br>2-Oxocarboxylic acid metabolism
37	5-Keto-DGluconicAcid_exchange	✓	present in ecocyc			
38	D-Fructose 6-Phosphate_exchange	✓	present in ecocyc			Methane metabolism
39	D-Glucose 1-Phosphate_exchange	✓	present in ecocyc			Glycolysis / Gluconeogenesis<br>Pentose and glucuronate interconversions

						Galactose metabolism<br>Starch and sucrose metabolism<br>Amino sugar and nucleotide sugar metabolism
40	Glyoxilic Acid_exchange	✓	present in ecocyc			Purine metabolism<br>Glycine, serine and threonine metabolism<br>Arginine and proline metabolism<br>Glyoxylate and dicarboxylate metabolism<br>Methane metabolism
41	Propionic Acid_exchange	✓	present in ecocyc			Propanoate metabolism<br>Ethylbenzene degradation<br>Nicotinate and nicotinamide metabolism
42	Thymine_exchange	✓	present in ecocyc			Pyrimidine metabolism
43	D-Malic Acid_exchange	✓	present in ecocyc			Butanoate metabolism
44	L-Galactonic acid, gamma-lactone_exchange					Ascorbate and aldarate metabolism
45	Alpha-Hydroxybutyric Acid_exchange					Propanoate metabolism
46	D-Galactonic acid, gamma-lactone_exchange		present in ecocyc			Galactose metabolism
47	D-Amino-N-ValericAcid_exchange					Lysine degradation<br>Arginine and proline metabolism
48	Dextrin_exchange					Starch and sucrose metabolism
49	L-Lyxose_exchange	✓	present in ecocyc			Pentose and glucuronate interconversions
50	M-Tartaric acid_exchange					Glyoxylate and dicarboxylate metabolism
51	β-Methyl-DGalactoside_exchange		present in ecocyc			
52	so3_exchange	✓	present in ecocyc			Cysteine and methionine metabolism<br>Taurine and hypotaurine metabolism<br>Sulfur metabolism
53	h2s_exchange	✓				Cysteine and methionine metabolism<br>Sulfur metabolism
54	Methyl-2-alpha-L-fucopyranosyl-beta-D-galactoside_exchange					
55	5-Oxopentanoate_exchange					Lysine degradation

Table S4. Predictions of GrowMatch.

	rxn ID	Presence in *i*JO1366	*E. coli* genes in KEGG or Ecocyc	BLASTP E value and gene ID of best hit	orphan reactions	KEGG pathway
1	R09079					Arginine and proline metabolism
2	R00904		b1444			beta-Alanine metabolism
3	R09081					Arginine and proline metabolism
4	R09077					Arginine and proline metabolism<br>beta-Alanine metabolism
5	R10338			4e-58 (b0121)		
6	R10347			3e-32 (b2937)		
7	R07226					

8	R00397					Alanine, aspartate and glutamate metabolism (Biosynthesis of amino acids)
9	R00357		b2574			Alanine, aspartate and glutamate metabolism
10	R07165					
11	R00400				✓	Alanine, aspartate and glutamate metabolism
12	R01713					Vitamin B6 metabolism
13	R07164					Nicotinate and nicotinamide metabolism
14	R00373				✓	
15	R00695				✓	
16	R00175					
17	R00265					
18	R01879				✓	
19	R00709		b1136			Citrate cycle<br>2-Oxocarboxylic acid metabolism
20	R07390		b0789/b1249			Glycerophospholipid metabolism
21	R01469			9e-71 (b2451)		
22	R01393				✓	Glyoxylate and dicarboxylate metabolism
23	R00825			4e-38 (b2538)		Aminobenzoate degradation
24	R02665					Tryptophan metabolism
25	R00818					Dioxin degradation<br>Polycyclic aromatic hydrocarbon degradation<br>Naphthalene degradation
26	R00823			4e-38 (b2538)		Aminobenzoate degradation
27	R01627					Phenylalanine, tyrosine and tryptophan biosynthesis
28	R00985		b1263/b1264			Phenylalanine, tyrosine and tryptophan biosynthesis
29	R06603					
30	R05539					
31	R04293					Tryptophan metabolism
32	R07803					Polycyclic aromatic hydrocarbon degradation
33	R09517					Tryptophan metabolism
34	R00157			1e-95 (b0474)		Purine metabolism
35	R00659		b1676/b1854			Glycolysis / Gluconeogenesis<br>Purine metabolism<br>Pyruvate metabolism
36	R00516		b2066			Pyrimidine metabolism
37	R00159					Pyrimidine metabolism
38	R00967		b2066			Pyrimidine metabolism
39	R00769		b1723/b3916			Glycolysis / Gluconeogenesis<br>Pentose phosphate pathway<br>Fructose and mannose metabolism<br>Galactose metabolism<br>Methane metabolism
40	R00287		b2781			Pyrimidine metabolism<br>Starch and sucrose metabolism
41	R03238		b1723/b3916			Galactose metabolism

42	R02096		b2066			Pyrimidine metabolism
43	R02097		b2066			Pyrimidine metabolism
44	R02095					Pyrimidine metabolism
45	R08515					
46	R00951					Starch and sucrose metabolism
47	R08946					Starch and sucrose metabolism
48	R02755					Lysine biosynthesis
49	R04336					Lysine biosynthesis
50	R00484					Alanine, aspartate and glutamate metabolism
51	R00822					Benzoate degradation
52	R00915					
53	R01797		b3918			Glycerophospholipid metabolism
54	R02030		b0789/b1249			Glycerophospholipid metabolism
55	R01799		b0175/b1409			Glycerophospholipid metabolism
56	R02027					Glycerophospholipid metabolism
57	R02057					Glycerophospholipid metabolism
58	R02051					Glycerophospholipid metabolism
59	R01800		b2585			Glycine, serine and threonine metabolism<br>Glycerophospholipid metabolism
60	R01801		b1912			Glycerophospholipid metabolism
61	R04176	✓			✓	
62	Gcald_exchange		present in ecocyc			Pentose and glucuronate interconversions<br>Glyoxylate and dicarboxylate metabolism<br>Vitamin B6 metabolism<br>Folate biosynthesis
63	ASPT		present in ecocyc			Alanine, aspartate and glutamate metabolism
64	AKGDH		present in ecocyc			Citrate cycle
65	ANS		present in ecocyc			Phenylalanine, tyrosine and tryptophan biosynthesis
66	NTPP7		present in ecocyc			Pyrimidine metabolism
67	NTPP8		present in ecocyc			Pyrimidine metabolism
68	GLCP		present in ecocyc			Glycolysis/Gluconeogenesis
69	ORNTA					Arginine and proline metabolism

Table S5. Predictions of GapFind/GapFill.

	**rxn ID**	**Presence in *i*JO1366**	***E. coli* genes in KEGG or Ecocyc**	**BLASTP E value and gene ID of best hit**	**orphan reactions**	**KEGG pathways**
1	R01078	✓	b0775			Biotin metabolism
2	R09396					Methane metabolism
3	R01377			1e-20 (b3671)		Phenylalanine metabolism
4	R01297				✓	Benzoate degradation

5	R07228					
6	R07598					
7	R10699			3e-78 (b0774)		Biotin metabolism
8	R00604			2e-50 (b0608)		Methane metabolism
9	R09498				✓	Sulfur metabolism
10	R10203					Sulfur metabolism
11	R10206	✓	b0935/b0937			Sulfur metabolism
12	R00699					Phenylalanine metabolism
13	R01325	✓	b0118/b0771/b1276			Citrate cycle<br>Glyoxylate and dicarboxylate metabolism
14	R02244	✓			✓	
15	R10848		b1580			Pentose and glucuronate interconversions
16	R02640			2e-22 (b1395)		Pentose and glucuronate interconversions
17	R01184					Ascorbate and aldarate metabolism<br>Inositol phosphate metabolism
18	R10866	✓	b1378			Pyruvate metabolism
19	R05188					Fatty acid biosynthesis
20	R01406	✓	b2836			Fatty acid degradation
21	R10123					Biotin metabolism
22	R10124	✓	b0776			Biotin metabolism
23	R00088	✓			✓	
24	R01299				✓	Benzoate degradation
25	R06895	✓	b2955/b3867			Porphyrin and chlorophyll metabolism
26	R10285			2e-138 (b3951)		
27	R00457			5e-37 (b2662)		
28	R09513					Sulfur metabolism
29	R01900	✓	b0118/b0771/b1276			Citrate cycle<br>Glyoxylate and dicarboxylate metabolism<br>2-Oxocarboxylic acid metabolism
30	R01481			2e-43 (b0608)		Pentose and glucuronate interconversions<br>Ascorbate and aldarate metabolism
31	R10859	✓	b2515			Terpenoid backbone biosynthesis
32	R00961					Purine metabolism
33	R05133		b1734			Glycolysis / Gluconeogenesis
34	R02736		b1852			Pentose phosphate pathway<br>Glutathione metabolism
35	R00453				✓	
36	R01216		b0134			Pantothenate and CoA biosynthesis
37	R00446					Tropane, piperidine and pyridine alkaloid biosynthesis
38	R09093					
39	R06862			2e-147 (b2935)		Methane metabolism
40	R05704					Cyanoamino acid metabolism
41	gbbtn_exchange	✓	present in Ecocyc			Lysine degradation

42	selnp_exchange					Selenocompound metabolism
43	crn_exchange	✓	present in Ecocyc			Bile secretion
44	crncoa_exchange					
45	btnso_exchange		present in Ecocyc			Biotin metabolism
46	ctbt_exchange	✓	b4111			
47	apoACP_exchange					Pantothenate and CoA biosynthesis
48	ctbtcoa_exchange					
49	bbtcoa_exchange					
50	seln_exchange					Selenocompound metabolism
51	HETZK		present in Ecocyc			Thiamine metabolism
52	GPDDA1					Glycerophospholipid metabolism
53	HMPK1		present in Ecocyc			Thiamine metabolism
54	ADOCBLS		present in Ecocyc			Porphyrin and chlorophyll metabolism
55	NNDMBRT		present in Ecocyc			Porphyrin and chlorophyll metabolism
56	RZ5PP		present in Ecocyc			Porphyrin and chlorophyll metabolism
57	CINNDO		present in Ecocyc			Phenylalanine metabolism
58	DHCIND		present in Ecocyc			Phenylalanine metabolism
59	PGLYCP		present in Ecocyc			Glyoxylate and dicarboxylate metabolism
60	GP4GH		present in Ecocyc			Purine metabolism
61	2DGLCNRx		present in Ecocyc			Pentose phosphate pathway
62	KG6PDC		present in Ecocyc			Pentose and glucuronate interconversions<br>Ascorbate and aldarate metabolism
63	X5PL3E		present in Ecocyc			Pentose and glucuronate interconversions<br>Ascorbate and aldarate metabolism
64	ADOCBIK		present in Ecocyc			Porphyrin and chlorophyll metabolism
65	ACBIPGT		present in Ecocyc			Porphyrin and chlorophyll metabolism
66	BETALDx		present in Ecocyc			Glycine, serine and threonine metabolism
67	AP4AH		present in Ecocyc			Purine metabolism
68	PLIPA3		present in Ecocyc			Glycerophospholipid metabolism
69	LPLIPA5					Glycerophospholipid metabolism
70	SPODM		present in Ecocyc			
71	GPDDA5					Glycerophospholipid metabolism
72	MI1PP		present in Ecocyc			Inositol phosphate metabolism
73	AB6PGH		present in Ecocyc			Glycolysis / Gluconeogenesis
74	DXYLK		present in Ecocyc			Pentose and glucuronate interconversions
75	DKGLCNR2y		present in Ecocyc			Pentose phosphate pathway
76	PEAMNO		present in Ecocyc			Phenylalanine metabolism
77	AP5AH		present in Ecocyc			Purine metabolism
78	GPDDA3					Glycerophospholipid metabolism
79	2DGLCNRy		present in Ecocyc			Pentose phosphate pathway
80	BETALDy		present in Ecocyc			Glycine, serine and threonine metabolism

81	LPLIPA3					Glycerophospholipid metabolism
82	DKGLCNR2x		present in Ecocyc			Pentose phosphate pathway
83	2DGULRx		present in Ecocyc			
84	2DGULRy		present in Ecocyc			

3- Mass-balancing of the KEGG dataset

KEGG has mass balance problems and we tried to resolve some of these problems. First, we found mass-imbalanced reactions using "checkMassChargeBalance" function in COBRA toolbox. Many imbalanced reactions are labeled as "incomplete" or "unclear" in KEGG. We removed such reactions. In case of imbalanced reactions in which macromolecules are broken down into their corresponding monomers, we removed the macromolecule from one side of the equation, as shown in the following example:

Macromolecule + h_2o -> monomer + Macromolecule

is replaced by:

Macromolecule + h_2o -> monomer

We should note that these kinds of replacements have also been done in iJR904[38] and *i*JO1366[39]. Finally, hydrogen was balanced in reactions in which all of the metabolites have known chemical formulas.

4- Robustness analysis with random reaction removals

In the manuscript, we investigated the sensitivity of GAUGE to the lack of GPRs and observed that GAUGE predictions are not significantly affected by the varying degrees of coverage of the GPRs. As another robustness analysis, we tried to see if we randomly remove reactions from the model, what percentage of them could be returned back using GAUGE. We applied GAUGE to iAF1260 *E. coli* model [40] to address this issue. We removed randomly 10 percent of the reactions from the model in 100 iterations. Each time, we added the removed reactions to the universal database and performed GAUGE to see how many reactions are predicted to be added back to the model. We observed that, on average, about 3 percent of the removed reactions are among the reactions that are predicted for addition to the model. Note that in order to improve GAUGE predictions, the removed reactions must generate new fully coupled reaction pairs, since GAUGE only analyzes fully coupled reaction pairs. Therefore, in a second attempt and in order to increase the probability of having new fully coupled reaction pairs in the model, we removed reactions with considering the following condition: If there are two reactions that are directionally coupled to a third reaction, we remove at most one of them in each iteration of generating random networks. Again, we observed that even in this case, GAUGE would again return only about 3 percent of removed reactions to the model. To explain this, we should note that after reaction removals in our second round of generating reduced networks, we introduced, on average, 290 new fully coupled reaction pairs to the model. However, only 30 pairs have "low" co-expressions according to the gene expression dataset. Hence, the second point that explains the low percentage is that GAUGE will only consider fully coupled reaction pairs with low gene co-expressions, for further analysis. We also performed the analysis on iJR904 and we observed that the percentage of returned reactions is less than 1%. As it is stated in the manuscript, directionally coupled or uncoupled reaction pairs may exist with high co-expression and we are not considering them as inconsistency cases. Therefore, converting these reaction pairs to fully coupled pairs will not generate a new inconsistency case to be considered in GAUGE.

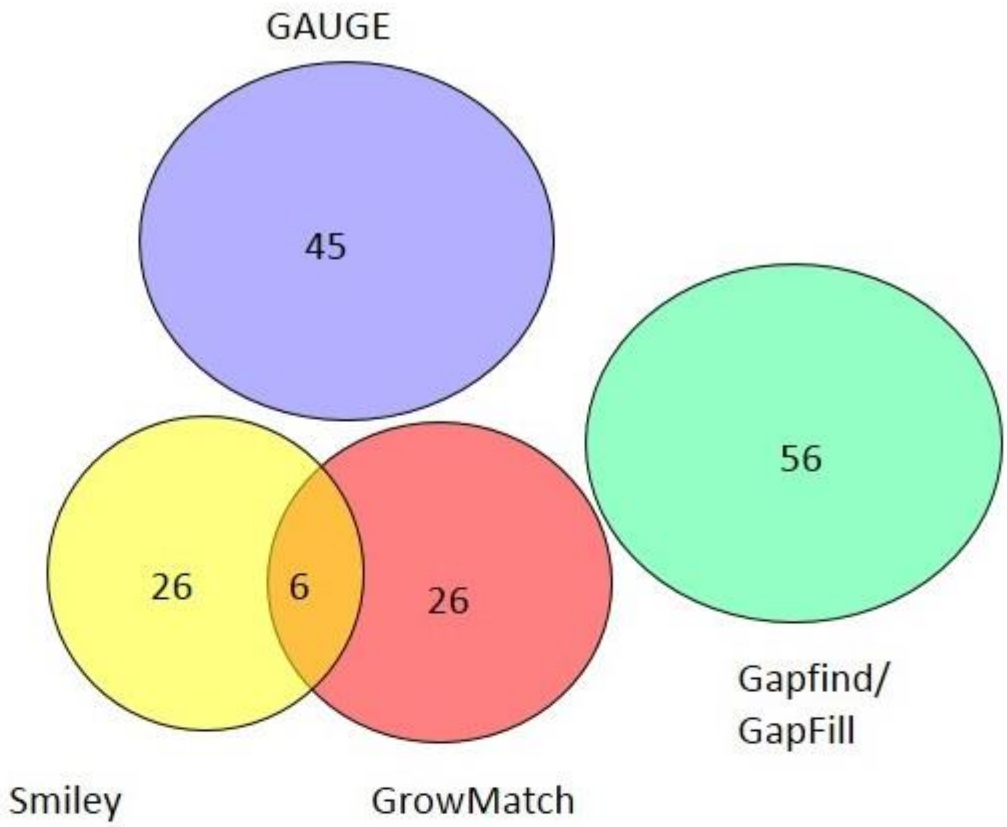

Figure S1. Venn diagram of reactions predicted by each method, when only positively validated reactions are considered

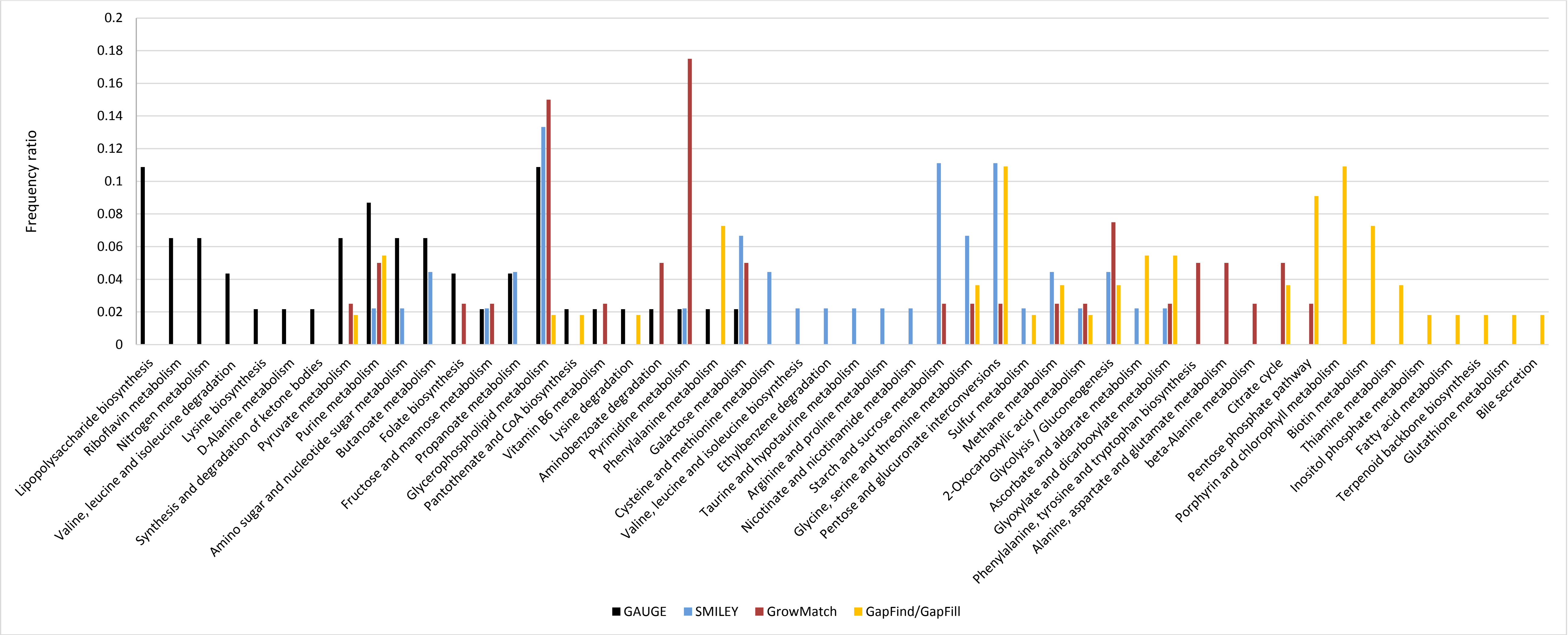

Figure S2. The frequency ratios of KEGG pathways in which the predicted gap filling reactions of each method are involved.

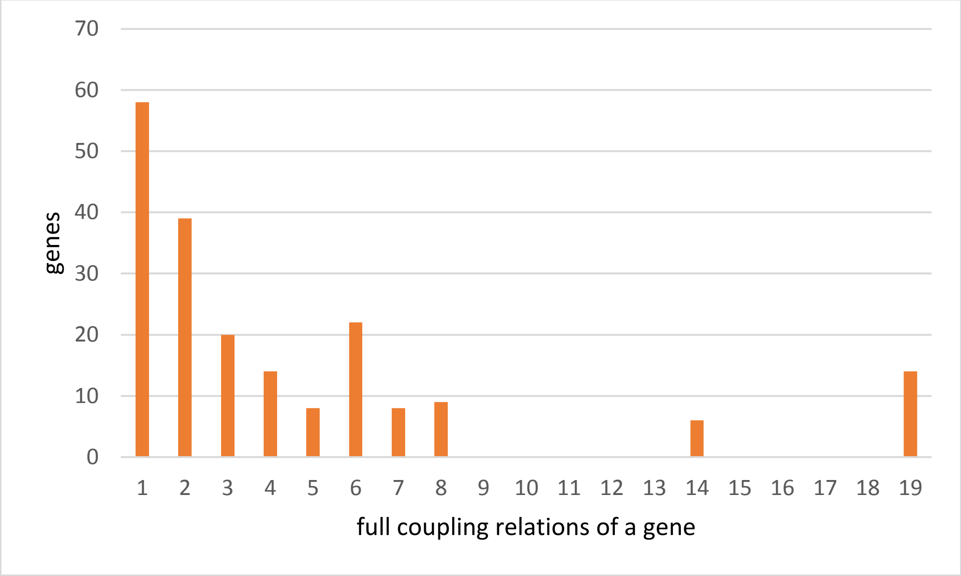

Figure S3. Number of genes which are involved in different number of full coupling relations

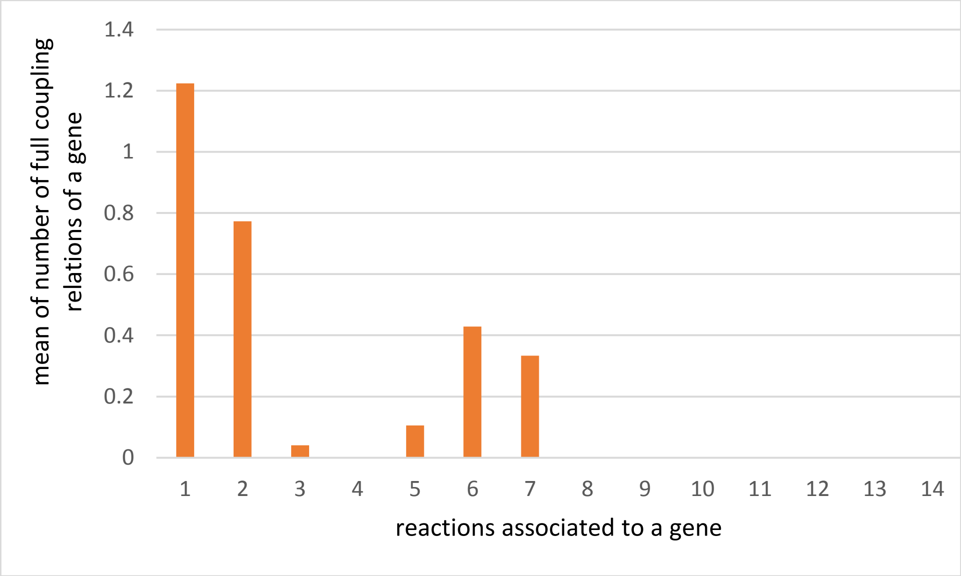

Figure S4. Relation between the number of full coupling relations and the number of associated reactions of a given gene

References

1 Katagiri, H., Yamada, H. & Imai, K. On the transphosphorylation reactions catalyzed by glucose-i-phosphate phosphotransferase of *Escherichia coli* i. enzymatic phosphorylation of riboflavin. *Journal of Biochemistry* **46**, 1119-1126 (1959).

2 Cohen, S. S. Utilization of gluconate and glucose in growing and virus-infected *Escherichia coli*. *Nature* **168**, 746-747 (1951).

3 Wong, C. H., Sugai, T. & Shen, G. J. (Google Patents, 1999).

4 Fischl, A. S. & Kennedy, E. P. Isolation and properties of acyl carrier protein phosphodiesterase of *Escherichia coli*. *Journal of Bacteriology* **172**, 5445-5449 (1990).

5 Thomas, J. & Cronan, J. E. The Enigmatic Acyl Carrier Protein Phosphodiesterase of *Escherichia coli* genetic and enzymological characterization. *Journal of Biological Chemistry* **280**, 34675-34683 (2005).

6 Beechey, R. & Happold, F. C. Pyridoxamine phosphate transaminase. *Biochemical Journal* **66**, 520 (1957).
7 Schell, U., Wohlgemuth, R. & Ward, J. M. Synthesis of pyridoxamine 5′-phosphate using an MBA: pyruvate transaminase as biocatalyst. *Journal of Molecular Catalysis B: Enzymatic* **59**, 279-285 (2009).
8 Saikusa, T., Rhee, H.-i., Watanabe, K., Murata, K. & Kimura, A. Metabolism of 2-oxoaldehydes in bacteria: purification and characterization of methylglyoxal reductase from *Escherichia coli*. *Agricultural and Biological Chemistry* **51**, 1893-1899 (1987).
9 Trent, M. S. Biosynthesis, transport, and modification of lipid A. *Biochemistry and Cell Biology* **82**, 71-86 (2004).
10 Opiyo, S. O., Pardy, R. L., Moriyama, H. & Moriyama, E. N. Evolution of the Kdo2-lipid A biosynthesis in bacteria. *BMC Evolutionary Biology* **10**, 362 (2010).
11 Skjold, A. C. & Ezekiel, D. H. Analysis of lambda insertions in the fucose utilization region of *Escherichia coli* K-12: use of lambda fuc and lambda argA transducing bacteriophages to partially order the fucose utilization genes. *Journal of Bacteriology* **152**, 120-125 (1982).
12 Schellenberger, J. *et al.* Quantitative prediction of cellular metabolism with constraint-based models: the COBRA Toolbox v2. 0. *Nature Protocols* **6**, 1290-1307 (2011).
13 Morgan, P. M., Sala, R. F. & Tanner, M. E. Eliminations in the reactions catalyzed by UDP-N-acetylglucosamine 2-epimerase. *Journal of the American Chemical Society* **119**, 10269-10277 (1997).
14 Sala, R. F., Morgan, P. M. & Tanner, M. E. Enzymatic formation and release of a stable glycal intermediate: the mechanism of the reaction catalyzed by UDP-N-acetylglucosamine 2-epimerase. *Journal of the American Chemical Society* **118**, 3033-3034 (1996).
15 Viswanathan, V., Green, J. M. & Nichols, B. P. Kinetic characterization of 4-amino 4-deoxychorismate synthase from *Escherichia coli*. *Journal of Bacteriology* **177**, 5918-5923 (1995).
16 Ziebart, K. T. & Toney, M. D. Nucleophile specificity in anthranilate synthase, aminodeoxychorismate synthase, isochorismate synthase, and salicylate synthase. *Biochemistry* **49**, 2851-2859 (2010).
17 Richey, D. P. & Brown, G. M. The biosynthesis of folic acid IX. Purification and properties of the enzymes required for the formation of dihydropteroic acid. *Journal of Biological Chemistry* **244**, 1582-1592 (1969).
18 Firestine, S. M., Poon, S.-W., Mueller, E. J., Stubbe, J. & Davisson, V. J. Reactions catalyzed by 5-aminoimidazole ribonucleotide carboxylases from *Escherichia coli* and *Gallus gallus*: a case for divergent catalytic mechanisms? *Biochemistry* **33**, 11927-11934 (1994).
19 Meyer, E., Leonard, N., Bhat, B., Stubbe, J. & Smith, J. Purification and characterization of the purE, purK, and purC gene products: identification of a previously unrecognized energy requirement in the purine biosynthetic pathway. *Biochemistry* **31**, 5022-5032 (1992).
20 Agarwal, R., Burley, S. K. & Swaminathan, S. Structural analysis of a ternary complex of allantoate amidohydrolase from *Escherichia coli* reveals its mechanics. *Journal of Molecular Biology* **368**, 450-463 (2007).
21 Serventi, F. *et al.* Chemical basis of nitrogen recovery through the ureide pathway: formation and hydrolysis of S-ureidoglycine in plants and bacteria. *ACS Chemical Biology* **5**, 203-214 (2010).
22 Werner, A. K., Romeis, T. & Witte, C.-P. Ureide catabolism in *Arabidopsis thaliana* and *Escherichia coli*. *Nature Chemical Biology* **6**, 19-21 (2010).
23 Percudani, R., Carnevali, D. & Puggioni, V. Ureidoglycolate hydrolase, amidohydrolase, lyase: how errors in biological databases are incorporated in scientific papers and vice versa. *Database* **2013**, bat071 (2013).
24 Bhaskar, V. *et al.* Identification of biochemical and putative biological role of a xenolog from *Escherichia coli* using structural analysis. *Proteins: Structure, Function, and Bioinformatics* **79**, 1132-1142 (2011).
25 Altaras, N. E. & Cameron, D. C. Metabolic engineering of a 1, 2-propanediol pathway in *Escherichia coli*. *Applied and Environmental Microbiology* **65**, 1180-1185 (1999).
26 Subedi, K. P., Kim, I., Kim, J., Min, B. & Park, C. Role of GldA in dihydroxyacetone and methylglyoxal metabolism of *Escherichia coli* K12. *FEMS Microbiology Letters* **279**, 180-187 (2008).
27 Doi, O. & Nojima, S. Lysophospholipase of *Escherichia coli*. *Journal of Biological Chemistry* **250**, 5208-5214 (1975).
28 Karasawa, K. *et al.* Purification and characterization of lysophospholipase L2 of *Escherichia coli* K-12. *Journal of Biochemistry* **98**, 1117-1125 (1985).
29 Mengin-Lecreulx, D., Flouret, B. & van Heijenoort, J. Pool levels of UDP N-acetylglucosamine and UDP N-acetylglucosamine-enolpyruvate in *Escherichia coli* and correlation with peptidoglycan synthesis. *Journal of Bacteriology* **154**, 1284-1290 (1983).

30 Samuel, J. & Tanner, M. E. Active site mutants of the "non-hydrolyzing" UDP-N-acetylglucosamine 2-epimerase from *Escherichia coli*. *Biochimica et Biophysica Acta (BBA)-Proteins and Proteomics* **1700**, 85-91 (2004).

31 Misra, K., Banerjee, A. B., Ray, S. & Ray, M. Glyoxalase III from *Escherichia coli*: a single novel enzyme for the conversion of methylglyoxal into D-lactate without reduced glutathione. *Biochem. J* **305**, 999-1003 (1995).

32 Subedi, K. P., Choi, D., Kim, I., Min, B. & Park, C. Hsp31 of *Escherichia coli* K-12 is glyoxalase III. *Molecular Microbiology* **81**, 926-936 (2011).

33 Passariello, C. *et al.* Biochemical characterization of the class B acid phosphatase (AphA) of *Escherichia coli* MG1655. *Biochimica et Biophysica Acta (BBA)-Proteins and Proteomics* **1764**, 13-19 (2006).

34 Baldoma, L. & Aguilar, J. Involvement of lactaldehyde dehydrogenase in several metabolic pathways of *Escherichia coli* K12. *Journal of Biological Chemistry* **262**, 13991-13996 (1987).

35 Anderson, P. M., Johnson, W. V., Endrizzi, J. A., Little, R. M. & Korte, J. J. Interaction of mono-and dianions with cyanase: evidence for apparent half-site binding. *Biochemistry* **26**, 3938-3943 (1987).

36 Walsh, M. A., Otwinowski, Z., Perrakis, A., Anderson, P. M. & Joachimiak, A. Structure of cyanase reveals that a novel dimeric and decameric arrangement of subunits is required for formation of the enzyme active site. *Structure* **8**, 505-514 (2000).

37 Parales, R. E. & Ingraham, J. L. The surprising Rut pathway: an unexpected way to derive nitrogen from pyrimidines. *Journal of Bacteriology* **192**, 4086-4088 (2010).

38 Reed, J. L., Vo, T. D., Schilling, C. H. & Palsson, B. O. An expanded genome-scale model of *Escherichia coli* K-12 (iJR904 GSM/GPR). *Genome Biol* **4**, R54 (2003).

39 Orth, J. D. *et al.* A comprehensive genome-scale reconstruction of *Escherichia coli* metabolism—2011. *Molecular Systems Biology* **7**, 535 (2011).

40 Feist, A. M. *et al.* A genome-scale metabolic reconstruction for Escherichia coli K-12 MG1655 that accounts for 1260 ORFs and thermodynamic information. *Molecular Systems Biology* **3**, 121 (2007).
